# Dual Framework for Classification and Detection of Third Molar Impaction in Panoramic Radiographs

**DOI:** 10.1016/j.identj.2026.109430

**Published:** 2026-02-09

**Authors:** Zohaib Khurshid, Mousa Haney Alsleem, Fahad Ahmed Aljubairah, Hussain Adel Alghafli, Abdullah Othman Alasafirah, Bassam Abbas Alibrahim, Abdullah Abdulrahman Alshamrani, Abdulelah Nasser Alsuhaymi

**Affiliations:** Department of Prosthodontics and Dental Implantology, College of Dentistry, King Faisal University, Al-Ahsa, Saudi Arabia

**Keywords:** Third molar impaction, Deep learning, YOLOv11n, ResNet50, Multihead self-attention, GAN augmentation, Surgical risk assessment

## Abstract

**Background:**

The surgical extraction of impacted mandibular third molars present significant clinical challenges, where accurate preoperative assessment is crucial to mitigate risks such as Inferior Alveolar Nerve injury. Although artificial intelligence shows promise in dental radiology, existing approaches are often limited to binary classification, affected by class imbalance, and lack standardized evaluation protocols, thereby restricting their clinical applicability.

**Methods:**

This study proposes two independent deep learning frameworks for comprehensive analysis of third molar impactions. The first framework is an end-to-end object detection pipeline employing modified YOLOv10 and YOLOv11n architectures enhanced with multihead self-attention. The second framework is a feature-based classification approach, where deep features extracted using ResNet50 and InceptionNetV3 are classified using traditional machine learning algorithms.

**Results:**

Validated on a multinational dataset of 5796 expertly annotated orthopantomograms with high inter-rater agreement (*κ* = 0.92), the proposed frameworks demonstrated competitive performance. The Fine KNN classifier using ResNet50 features achieved the best classification performance, yielding 97.56% accuracy, 96.07% precision, 96.21% recall, and an F1-score of 96.10%, while InceptionNetV3-based classification achieved 97.33% accuracy with an F1-score of 95.30%. For object detection, YOLOv11n attained a mean average precision of 88.9% (mAP@0.5) and 85.7% (mAP@0.5:0.95), while maintaining substantially lower computational complexity (19.7 vs 28.4 GFLOPs). Ablation experiments confirmed that the integration of multihead self-attention modules and generative adversarial network-based augmentation improved detection performance by 6.4% mean average precision.

**Conclusions:**

The proposed frameworks enable accurate and automated multiclass assessment of third molar impactions, achieving high diagnostic performance while preserving computational efficiency suitable for clinical deployment. This work advances artificial intelligence-assisted surgical planning by providing reliable F1-score-based evaluation, reliable real-time detection, and enhanced preoperative risk stratification in oral and maxillofacial surgery.

## Introduction

The surgical removal of third molars remains one of the most frequently performed procedures in oral and maxillofacial surgery, yet it continues to pose notable clinical and diagnostic challenges.^1^ Despite refinements in surgical technique, postoperative complications, particularly neurosensory deficits, are still widely reported.^2^ Permanent or long-lasting injury to the Inferior Alveolar Nerve (IAN) accounts for most severe adverse outcomes, with lower third molar extraction representing the primary etiological factor, outpacing implant placement and orthognathic interventions in incidence and severity.^3^ Such injuries manifest as persistent aesthesia, hypesthesia, paraesthesia, neuropathic pain, and, through their chronic burden, measurable psychological distress, including anxiety and depression.^4^ Orthopantomogram (OPG) radiographs remain the predominant modality for this risk assessment due to their accessibility, speed, and low radiation dose.^5^ Several radiographic signs root darkening, canal deflection, interruption of the lamina dura, and loss of cortication, serve as conventional predictors of intimate molar-canal relationships and increased IAN vulnerability.^6^, ^7^, ^8^ The degree of radiographic overlap between the tooth roots and the canal has been strongly correlated with injury risk in both retrospective and prospective cohorts.^9^, ^10^, ^11^ However, PRs provide only a two-dimensional representation of a three-dimensional anatomical interaction. Distortions related to patient positioning, superimpositions, variable grey-scale values, and inconsistent projection geometry limit the reliability of interpretation, particularly for novice clinicians who have not yet developed a nuanced image-reading experience.^12^^,^^13^ These limitations have opened the door for artificial intelligence (AI) and deep learning approaches to enhance diagnostic consistency and objectivity in preoperative evaluation.

In recent years, convolutional neural networks (CNNs) and associated deep learning architectures have reshaped the field of medical imaging through their capacity for hierarchical feature extraction and spatial contextualization^14^, ^15^, ^16^ Celik^17^ developed a two-stage framework incorporating Faster R-CNN and YOLOv3 and reported an impressive mAP@0.5 of 0.96; however, the model offered no angular classification and therefore limited its clinical usefulness for surgical risk estimation. Similarly, Güller et al^18^ compared Faster R-CNN and YOLOv3, finding YOLOv3 superior in precision and recall, and mAP@0.5 of 96%; recall of 93%, but once again, angular and positional subtype classification, central to operative planning, was not addressed. Similarly, Silva et al^19^ developed a semisupervised transformer-based model across 8795 OPGs, reaching performance comparable to junior specialists but without directly addressing impaction detection or subtype classification. Faadiya et al^20^ reported accuracy ranges of 78.9% to 90.2% across multiple AI systems for molar evaluation, highlighting persistent concerns about dataset heterogeneity and limited sample sizes, particularly for infrequently encountered impaction types. Another study by Chindanuruks et al^21^ showed that deep learning architectures outperform traditional CNNs for dental implant detection, reinforcing the potential of advanced networks but also emphasizing the importance of architecture choice, dataset diversity, and fine-tuning strategy. These overarching challenges continue to limit standardization, usability, and generalizability in third molar AI systems. Beyond technical performance, ethical and professional concerns add further dimensions. Tuygunov et al^15^ stressed that integrating AI into dental workflows requires robust validation frameworks, transparent architectures, and explicit risk-benefit analyses, particularly for complex, high-stakes diagnostic tasks such as impaction assessment. Many existing models lack this level of rigour, with datasets often limited to single-institution radiographs, insufficient annotation consensus, or unrepresentative demographic diversity. Together, these findings reveal a clear pattern: progress has been made in detection tasks, but significant gaps remain in multilabel impact classification, modelling of rare subtypes, mitigation of dataset imbalance, computational efficiency, and standardized evaluation. Moreover, most prior studies treat detection and classification as isolated subprocesses, neglecting the benefits of an integrated pipeline capable of learning contextual anatomical relationships end-to-end. This disconnect reduces clinical utility, especially for real-time applications where speed and robustness are essential.

This study responds to these challenges by developing a dual framework for detection and classification utilizing a multinational unannotated publicly available panoramic radiograph dataset from two different populations from the Mendeley dataset,^22^^,^^23^ annotated by final year undergraduate dental program students and dental interns. This annotation was verified by two dental specialists, Z.K. and A.A.F. After this annotation was enhanced through systematic preprocessing and augmentation. Unlike most prior work, the approach incorporates both deep CNN features of ResNet50 and InceptionV3 models and classifies by a suite of machine learning algorithms, enabling a more interpretable, high-level, and handcrafted feature representations. The detection pipeline leverages a modified YOLOv11n architecture enriched with multihead self-attention (MHSA) modules to enhance angular subtype discrimination, alongside a lightweight YOLOv10 variant optimized through genetic algorithms for real-time clinical deployment. Addressing class imbalance, particularly the underrepresentation of inverted impactions, this study applies generative adversarial networks (GANs) to expand minority class samples, promoting reliable learning across all subclasses. Through these contributions, the work advances a clinically interpretable, computationally efficient, and diagnostically comprehensive solution to a persistent challenge in oral surgery.

This study aims to develop and validate two independent deep learning frameworks for the automated assessment of mandibular third molar impactions from OPG. The research seeks to create a reliable, multiclass system for accurate impaction classification and real-time detection to support clinical decision-making. The objective is to achieve expert-level diagnostic performance while addressing critical challenges such as class imbalance and anatomical complexity, ultimately enhancing preoperative risk stratification in oral surgery.

This study proposes two independent analytical frameworks rather than two stages of a single pipeline. The first framework follows a feature-based classification paradigm, where deep features are extracted using pretrained CNNs such as ResNet50 and InceptionNetV3, and subsequently classified using traditional machine learning classifiers. The second framework is an end-to-end object detection approach based on YOLO architecture, which directly localizes and classifies impacted mandibular third molars from panoramic images without explicit feature extraction or separate classification stages.

## Methods

**Fig. 1:**
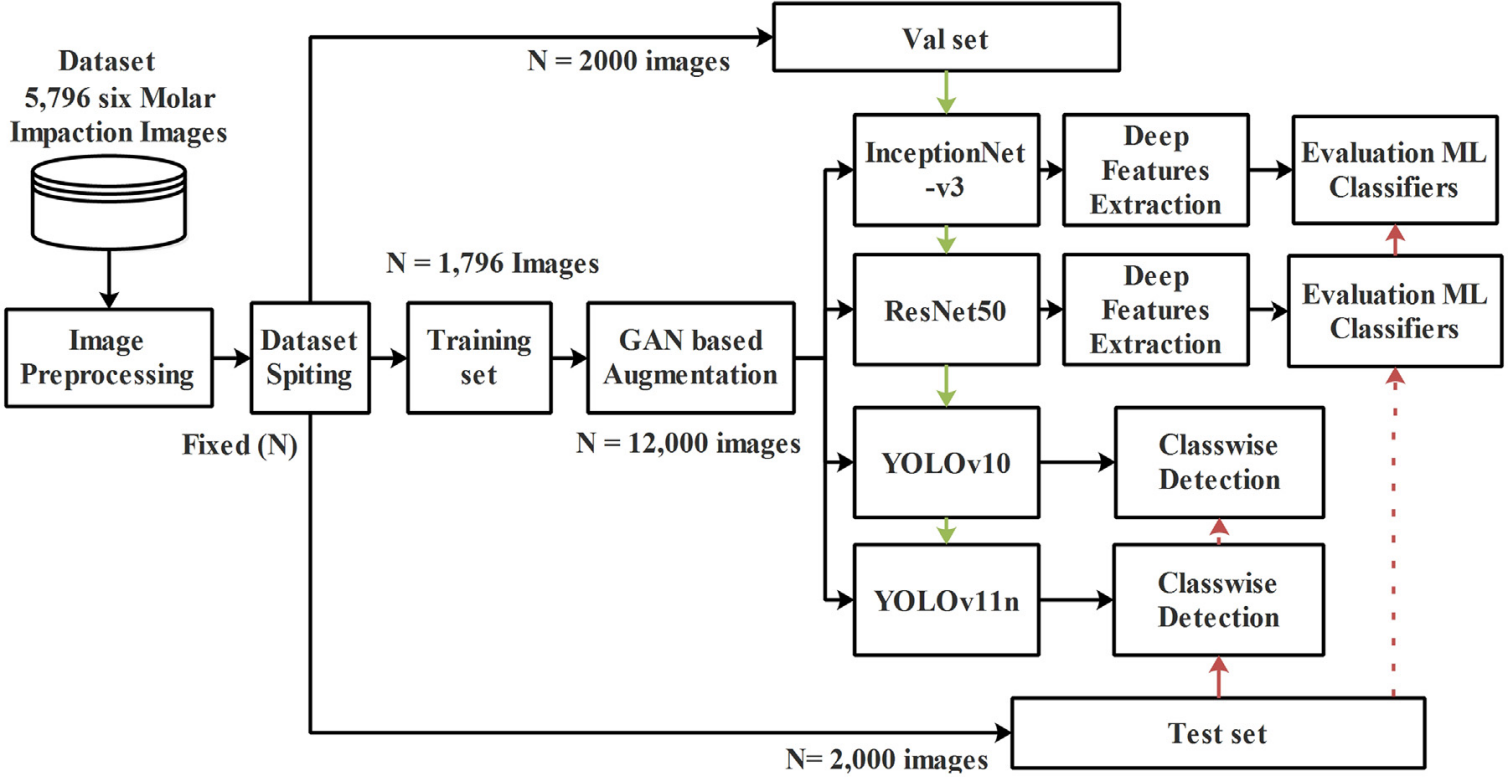
Overview of the proposed dual framework, illustrating independent deep feature extraction pipelines for classification and an end-to-end detection pipeline without feature fusion.

This study proposes two independent deep learning frameworks for third molar impaction analysis using an expert-annotated multinational dataset.^22^^,^^23^ The original, unannotated dataset was professionally labelled by undergraduate dental students and dental interns under the two dental specialists using the CVAT annotation tool to establish clinical ground truth for all six impaction classes; incomplete or corrupted images were excluded before model training, as shown in Figure 1. To mitigate class imbalance, the dataset was standardized to 2000 images per label through augmentation. The first framework is an end-to-end object detection pipeline employing modified YOLOv11n with MHSA and a genetically optimized YOLOv10 architecture for simultaneous localization and detection. The second is a distinct feature-based classification approach that leverages ResNet50 and InceptionV3 for deep feature extraction, followed by traditional machine learning classifiers. Both frameworks were developed and evaluated separately on comprehensively augmented data, providing clinicians with versatile options for automated impaction assessment. This dual-methodology design enables comprehensive benchmarking while addressing diverse clinical requirements for accuracy and computational efficiency, as AI guideline.^24^ This study follows the Checklist for Artificial Intelligence in Medical Imaging (CLAIM) 2024,^25^ to ensure methodological rigour and reproducibility (see Supplementary File-1).

### Ethical clearance

This study was conducted using a publicly available, fully anonymized dataset obtained from Mendeley Data.^22^^,^^23^^,^^26^ However, to ensure full compliance with institutional requirements, ethical approval for the project was formally granted by the Research Ethics Committee at King Faisal University under Reference No. KFU-REC-2025-SEP–ETHICS3540. The dataset is distributed under the Creative Commons Attribution 4.0 International (CC BY 4.0) license, which allows unrestricted use, distribution, and reproduction provided the original authors are properly cited. As no identifiable clinical or personal information was included and all imaging data were anonymized before public release, the study posed minimal ethical risk. The research was therefore deemed ethically acceptable in accordance with institutional and international guidelines.

### Inclusion criteria

Only OPG radiographs that clearly displayed the mandibular third molar region and allowed reliable identification of impaction patterns were included. Images had to be complete, high-resolution, and free from excessive artefacts or structural loss. Radiographs that corresponded to one of the six predefined impaction categories were retained for annotation and model development. Since this was a dataset-based study, no demographic or clinical participant criteria were involved.

### Exclusion criteria

Radiographs were excluded if they were partially missing anatomical structures, excessively blurred, or exhibited motion artefacts that hindered accurate visualization of the mandibular third molars. Images without a clear or classifiable impaction type were also removed. No patient-specific exclusion factors were required, as the dataset contained only anonymized panoramic radiographs without demographic information.

### Dataset annotation and standardization

The dataset, originally unannotated, was labelled by a group of experts as mentioned earlier in methodology using CVAT to identify bounding boxes and assign one of six impaction classes. All images were standardized to uniform dimensions, brightness, and contrast to ensure consistency between training samples. Inter-rater review confirmed high annotation reliability. Multiple preprocessing steps, including normalization and resizing for both detection and classification pipelines, were applied to maintain consistent radiographic quality across the dataset.

### Dataset collection

The dataset was sourced from a publicly available repository, Mendeley Data,^22^^,^^23^ and annotated into six distinct impact classes, comprising 5796 panoramic radiographs. All images were independently labelled by experienced dental specialists (authors) using the open-source computer vision annotation tool CVAT to ensure precise anatomical delineation. Inter-rater reliability was quantified using Cohen’s *κ* = 0.92 (*P* < .001), indicating excellent agreement among annotators. The dataset was divided using a fixed hold-out strategy into training (*n* = 1796), validation (*n* = 2000), and independent testing (*n* = 2000) set prior to augmentation. Cross-validation was not employed due to the large dataset size and the use of a dedicated test set for unbiased performance evaluation.

### Experimental environment

All models were developed and trained using a cloud-based computing environment to manage the computational demands of deep learning. The object detection architectures, YOLOv10 and YOLOv11n, were implemented on Google Colab utilizing an NVIDIA A100 GPU. The deep feature extraction and classification pipelines employing ResNet50 and InceptionNetV3 were executed on the Kaggle platform, leveraging an NVIDIA P100 GPU. This setup provided the necessary high-performance computing resources for efficient model training and validation.

### Data preprocessing and augmentation

**Fig. 2:**
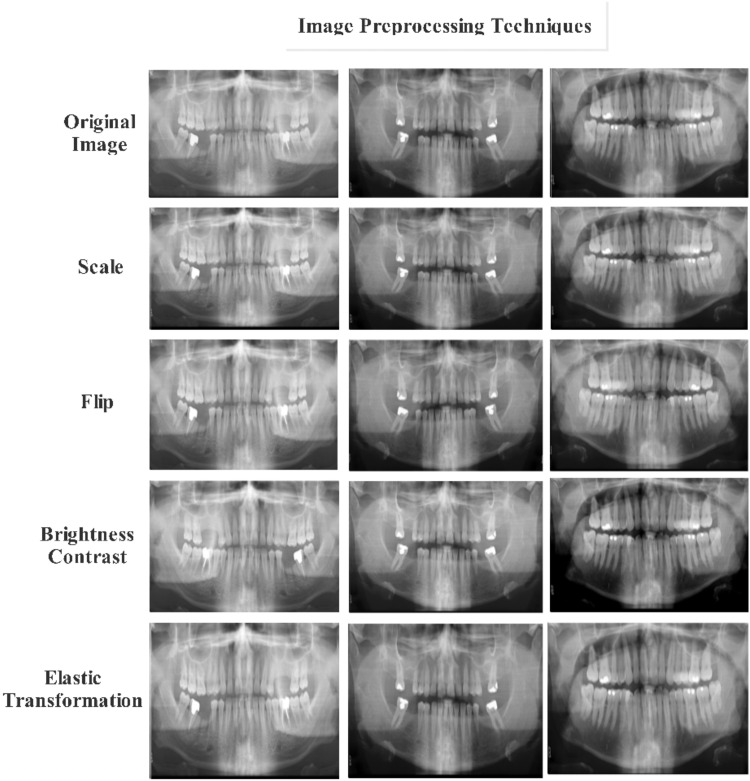
Enhance third molar impactions on OPGs with these image preprocessing techniques.

All panoramic radiographs underwent standard preprocessing to improve image quality and ensure consistency across the dataset. This included adjustments to orientation, brightness, contrast, and overall image clarity to account for variations commonly seen in clinical imaging. Basic geometric transformations were also applied to simulate natural variability in patient positioning, helping the models generalize to real-world radiographs, as shown in Figure 2.

To address class imbalance, particularly for rare impaction types such as inverted impactions, a comprehensive augmentation strategy was applied to the training set. This included a combination of geometric and photometric variations to increase the diversity of training samples and improve model robustness. Bounding boxes were carefully updated during augmentation to preserve anatomical accuracy.

For classes with very limited representation, additional synthetic images were generated using an advanced generative model such as StyleGAN2-ADA. This technique produced realistic and anatomically consistent radiographs that helped balance the dataset and prevented the models from becoming biased towards more common impaction types. All augmented and synthetic images were reviewed to ensure they were appropriate for use in training. The class-wise distribution of the dataset before and after augmentation is summarized in Table 1.

**Table 1:** Class distribution of third molar impactions.

Class ID	Class name	Instances	Training original	Validation original	Testing original	Augmentation training set
0	Distoangular	1470	470	500	500	2000
1	Mesioangular	1100	100	500	500	2000
2	Vertical	939	339	300	300	2000
3	Horizontal	817	217	300	300	2000
4	Buccolingual	734	334	200	200	2000
5	Inverted	736	336	200	200	2000
-	**Total**	**5796**	**1796**	**2000**	**2000**	**12,000**

### Model development

Two modern deep learning approaches were developed for automated third molar impaction analysis. The first approach focused on object detection using the latest YOLOv10 and YOLOv11n models. These architectures were selected because they provide a strong balance between accuracy and computational speed, making them suitable for potential real-time clinical use on panoramic radiographs.

#### Modified YOLOv11n architecture

The YOLOv11n model was adapted to improve the detection of subtle impaction patterns. MHSA modules were integrated into the neck of the YOLOv11n architecture to enhance contextual feature aggregation before the detection head, improving discrimination of angular impaction subtypes. This modification helped the model differentiate between closely related impaction types and improved performance across all six categories. The final system can localize impacted third molars and assign the appropriate subtype label in a single step, supporting rapid clinical decision-making.

#### Modified YOLOv10 architecture

The YOLOv10 model was also refined for this study. Similar attention enhancements were introduced to help the network interpret complex radiographic areas where third molars may be partially hidden or overlapped with surrounding structures. Additional adjustments were made to improve the model’s ability to use information from multiple scales within the radiograph. These refinements aimed to provide more consistent detection across different angulation patterns and patient variations. The final version maintained fast processing speeds suitable for chairside applications.

#### ResNet50 architecture

For the classification framework, ResNet50 was used as one of the primary feature extractors. This architecture is well-established in medical imaging and is known for its ability to capture both fine and broad radiographic details. When applied to panoramic radiographs, ResNet50 generated rich feature representations that supported accurate classification of all six impaction subtypes. The model was chosen for its reliability, interpretability, and strong performance in previous dental imaging research.

#### InceptionNetV3 architecture

InceptionNetV3 served as a second feature extractor for the classification pathway. Its design enables the model to evaluate features at multiple scales simultaneously, which is helpful when distinguishing between impaction types that differ in angulation or spatial orientation. It has been widely used in medical imaging tasks due to its efficiency and ability to capture subtle radiographic patterns. Together with ResNet50, it provided complementary feature information for the classification algorithms.

### Feature extraction for classification

Both feature extractors were applied to preprocessed panoramic radiographs to convert each image into a numerical representation containing clinically relevant information. These extracted features were then used as inputs to traditional machine learning classifiers. The Fine KNN classifier was implemented with *k* = 1 using Euclidean distance and standardized deep feature vectors extracted from ResNet50 and InceptionNetV3. The combination of deep-learning features and machine learning models enabled highly accurate identification of impaction subtypes while maintaining interpretability for clinical use.

### Performance metrics for dental impaction

These metrics quantitatively assess classification performance in third molar impaction analysis. Accuracy reflects overall classification correctness across all impaction classes. Precision is defined as the proportion of true positive predictions among all positive predictions, indicating the model’s tendency to limit false positives. Recall represents the proportion of true positive cases correctly identified among all actual positive instances. The F1-Score provides a harmonic balance between precision and recall and is particularly informative in multiclass settings where class-wise trade-offs exist. ROC AUC measures threshold-independent discriminative capability, supporting robustness across varying decision thresholds. All metric definitions follow standard statistical and machine learning conventions, while any clinical interpretations discussed in this study are contextual and specific to the application domain. In addition to accuracy, the F1-score is emphasized as a primary evaluation metric due to its balanced representation of classification performance across multiple classes. Classification results were computed using the standard performance metrics summarized in Table 2.

**Table 2:** Quantitative evaluation metrics.

Metric	Formula	Clinical interpretation
**Accuracy**	$\frac{TP+TN}{TP+TN+FP+FN}$	Overall diagnostic correctness in discriminating impaction phenotypes
**Precision**	$\frac{TP}{TP+FP}$	Reliability in identifying true impactions (minimizing false surgical referrals)
**Recall**	$\frac{TP}{TP+FN}$	Sensitivity in detecting occult impactions (reducing missed diagnoses)
**F1 score**	$2\times \frac{\text{Precision}\times \text{Recall}}{\text{Precision}+\text{Recall}}$	Balanced measure for class-imbalanced radiographic datasets
**ROC AUC**	Area under the ROC curve	Discrimination capacity across all decision thresholds

## Results

### Classification results

The evaluation of classifiers leveraging deep feature extractors demonstrated exceptional performance in multilabel third molar impaction classification, as shown in Table 3. Among all configurations, the Fine KNN classifier utilizing ResNet50 features achieved the highest performance, with an accuracy of 97.6%, precision of 96.1%, recall of 96.2%, and an F1-score of 96.1%. This was closely followed by Neural Networks and AdaBoost models, which also consistently yielded accuracy and F1-scores above 97.0% and 95.0%, respectively, across both ResNet50 and InceptionNetV3.

**Table 3:** Comprehensive evaluation of classifiers using ResNet50 and InceptionNetV3 feature extractors.

Classifier	Model	Train time(s)	Prediction time(s)	Accuracy (%)	Precision (%)	Recall (%)	F1 score (%)
**Neural network**	ResNet50	9.612	0.020	97.11	95.68	95.12	95.38
	InceptionNetV3	8.050	0.017	96.80	94.90	94.40	94.60
**Cubic SVM**	ResNet50	29.784	0.394	96.50	94.93	92.75	93.82
	InceptionNetV3	27.266	0.381	96.40	95.70	92.30	93.60
**Fine KNN**	ResNet50	0.027	0.003	97.56	96.07	96.21	96.10
	InceptionNetV3	0.025	0.002	97.33	95.30	95.40	95.30
**Logistic regression**	ResNet50	4.909	0.010	96.70	95.13	94.48	94.70
	InceptionNetV3	4.504	0.009	96.50	94.60	93.90	94.20
**Decision tree**	ResNet50	6.889	0.003	95.30	91.54	94.40	92.94
	InceptionNetV3	6.098	0.003	95.20	90.30	93.80	91.60
**Naive Bayes**	ResNet50	0.312	0.050	94.95	90.80	95.05	92.87
	InceptionNetV3	0.296	0.045	94.90	90.30	94.70	91.60
**Gradient boosting**	ResNet50	460.245	0.012	96.91	96.05	93.42	94.62
	InceptionNetV3	432.731	0.011	96.70	95.80	92.90	94.10
**Linear SVM**	ResNet50	4.238	0.030	96.81	95.18	94.63	94.90
	InceptionNetV3	3.864	0.029	96.60	94.70	94.00	94.20
**Random forest**	ResNet50	14.870	0.051	97.00	96.10	93.50	94.78
	InceptionNetV3	13.586	0.049	96.80	95.90	92.90	94.10
**AdaBoost**	ResNet50	89.750	0.102	97.16	96.04	94.03	95.02
	InceptionNetV3	85.244	0.099	96.90	95.80	93.70	94.60

**Fig. 3:**
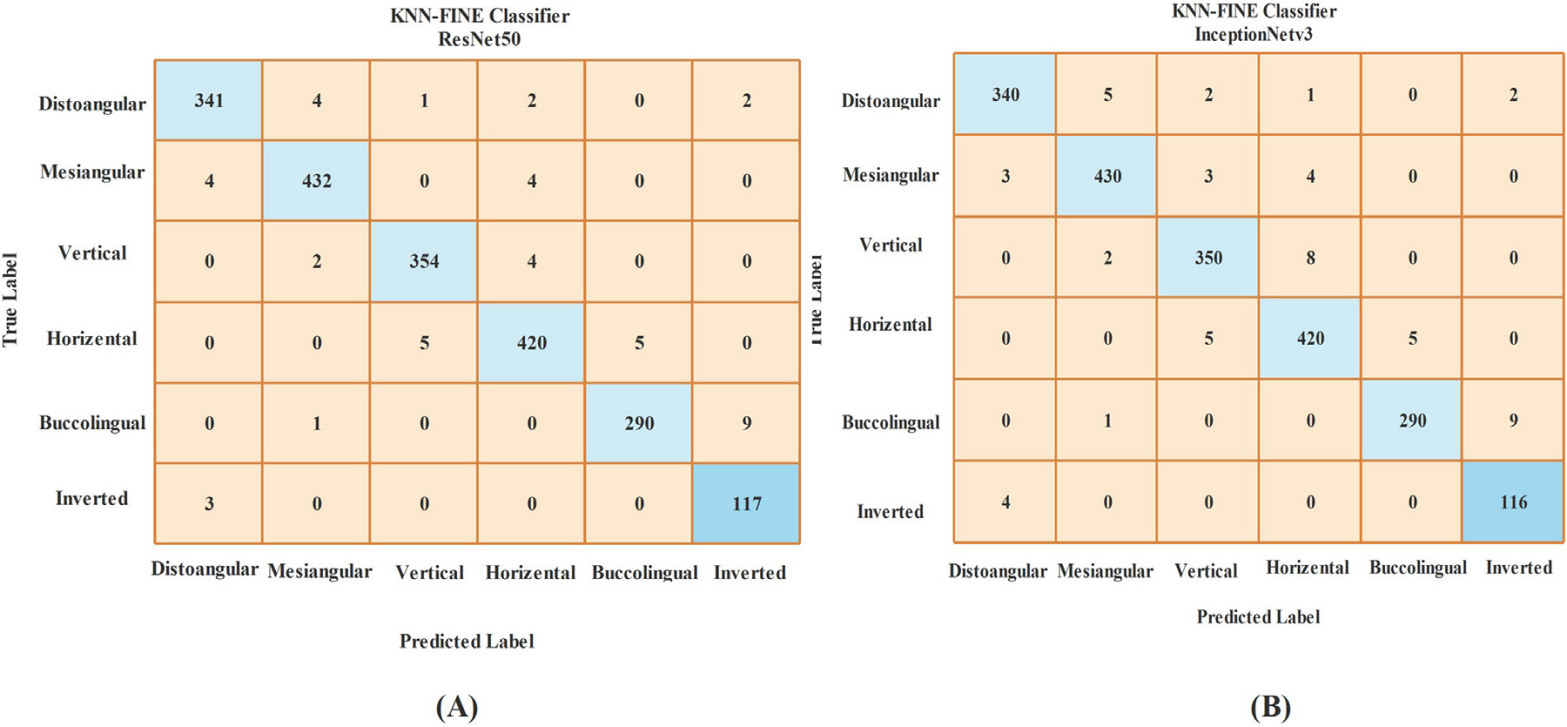
Fine KNN confusion matrix (A) for ResNet50 and (B) for InceptionNetv3 model.

A class-wise analysis of the two top-performing proposed classifiers is provided by their confusion matrices in Figure 3. The matrix for the Fine KNN model with ResNet50 features (A) shows high diagonal values across all impact categories, indicating consistently correct classifications. Similarly, the matrix for the Fine KNN model with InceptionNetV3 features (B) confirms robust performance, with the majority of predictions lying on the diagonal. Misclassifications were primarily observed between mesioangular and horizontal impactions, which exhibit strong morphological overlap in panoramic radiographs due to similar crown root angulation and 2D projection effects. Since all classes were balanced through augmentation as shown in Table 1, these errors are attributed to radiographic similarity rather than class imbalance.

### Detection results

The results for the proposed object detection-based approach are presented in Table 4. The modified YOLOv10 architecture achieved an overall mAP@0.5 of 87.60% with a recall of 93.10%, while the modified YOLOv11n model yielded a superior overall mAP@0.5 of 88.9% and a recall of 94.4%. Class-wise analysis revealed a notable performance pattern: both models exhibited exceptional precision and recall for Buccolingual and Inverted impactions, with mAP@0.5 scores exceeding 99.50%. In contrast, the Distoangular, Mesioangular, and Horizontal subtypes demonstrated high recall of 86% but notably lower precision, ranging from 62.40% to 75.20%.

**Table 4:** Performance of YOLO architectures for third molar impaction detection.

Class	Model	Precision (P)	Recall (R)	mAP@0.5	mAP@0.5:0.95	Parameters (M)	GFLOPs
**All (overall)**	YOLOv10	76.20	93.10	87.60	84.20	11.1	28.4
	YOLOv11n	78.20	94.40	88.90	85.70	8.9	19.7
**Distoangular**	YOLOv10	67.00	92.20	84.70	79.80	–	–
	YOLOv11n	68.80	93.20	86.10	81.60	–	–
**Mesioangular**	YOLOv10	62.40	86.00	80.70	74.80	–	–
	YOLOv11n	64.50	87.40	82.10	76.80	–	–
**Vertical**	YOLOv10	73.00	92.60	83.50	79.90	–	–
	YOLOv11n	75.20	93.60	84.90	81.10	–	–
**Horizontal**	YOLOv10	68.40	88.10	80.60	74.50	–	–
	YOLOv11n	70.70	89.20	82.10	76.20	–	–
**Buccolingual**	YOLOv10	86.30	99.80	96.90	96.66	–	–
	YOLOv11n	87.50	99.90	97.40	97.10	–	–
**Inverted**	YOLOv10	99.90	100.0	99.50	99.50	–	–
	YOLOv11n	100.0	100.0	99.60	99.60	–	–

**Fig. 4:**
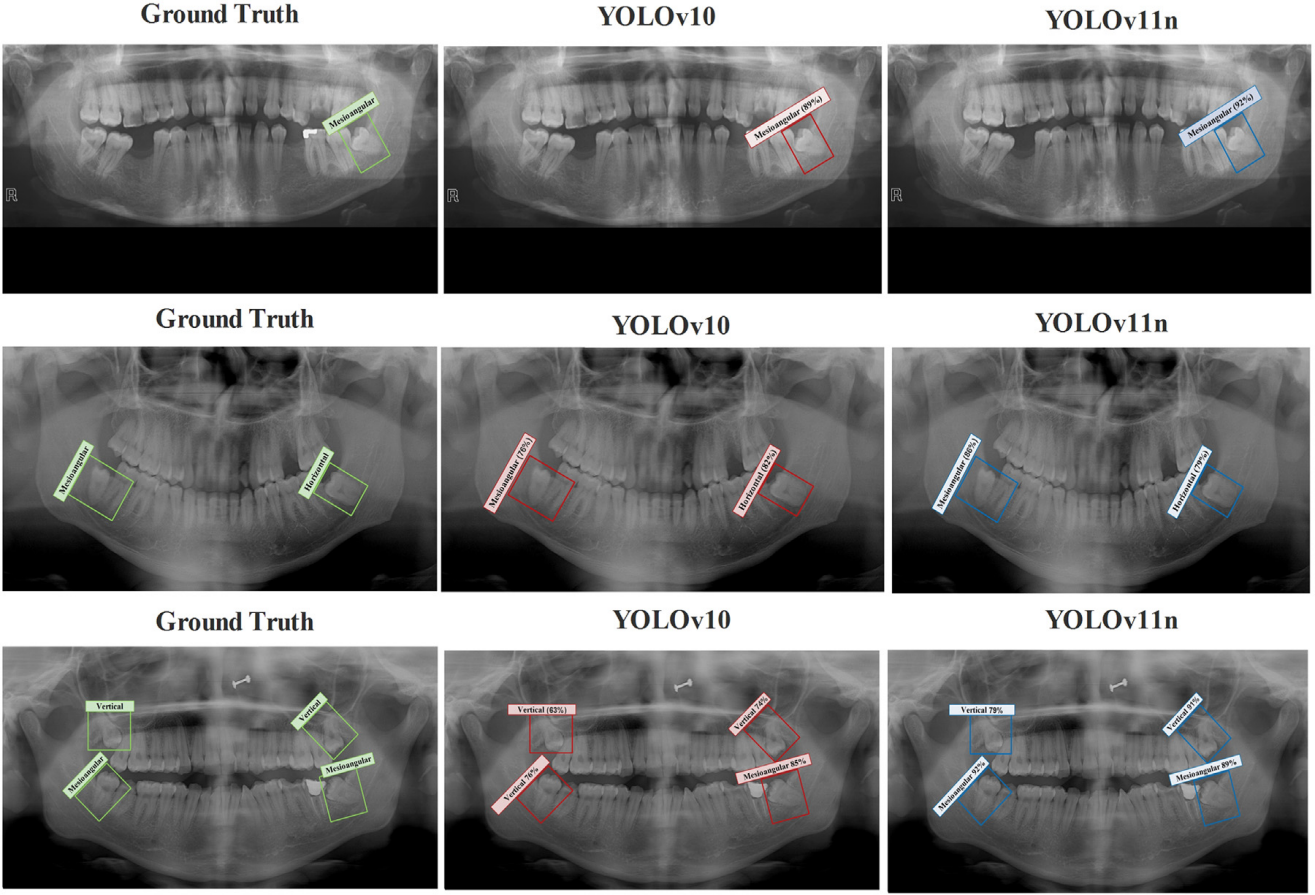
Representative test samples showing ground-truth labels and corresponding predictions produced by YOLOv10 and YOLOv11n.

The comparison between the actual ground-truth labels and the predicted labels generated by the YOLOv10 and YOLOv11n detection models. The figure shows how both models detect third molar impactions across test samples, highlighting differences in prediction accuracy. This visualization helps demonstrate the consistency and reliability of the detection frameworks, as shown in Figure 4.

### Statistical analysis

Model performance was assessed using commonly reported evaluation metrics, including accuracy, precision, recall, F1-score, and mean average precision (mAP). These measures allowed us to compare the effectiveness of different classification and detection models in identifying third molar impaction types. For the classification framework, results from all models were compared to determine which approach provided the most reliable performance across the six impaction categories. Confidence intervals were calculated to provide an estimate of the stability and consistency of each metric. For the detection framework, the performance of the two YOLO-based models was compared using mAP values across all classes. This comparison enabled us to identify which architecture offered more accurate localization and subtype detection of impacted third molars. The statistical analysis ensured that the observed performance differences between models were meaningful and reflected genuine improvements in diagnostic capability.

### Ablation study

An ablation study was conducted to quantify the contribution of each key component in the proposed framework. The baseline model YOLOv10 without MHSA or GAN augmentation achieved an mAP@0.5 of 82.1%. Integrating the MHSA mechanism provided a significant boost of +3.5%, elevating the mAP to 85.6% by enhancing the model’s focus on critical anatomical relationships. Subsequently, addressing the severe class imbalance through GAN-based synthesis for minority classes, particularly Inverted and Buccolingual, further improved the mAP by +2.9%, resulting in the final performance of 88.5%. This systematic evaluation confirms that both the MHSA module for contextual feature enrichment and the GAN-driven data balancing are indispensable for achieving state-of-the-art performance in this complex diagnostic task.

## Discussion

This study presents a comprehensive evaluation of dual deep learning frameworks for the automated detection and classification of third molar impactions. Our findings demonstrate that both proposed approaches, a deep feature-based classification pipeline and optimized YOLO-based detection architectures, achieve diagnostic performance suitable for clinical application. The classification framework yielded exceptional results. Specifically, the ResNet50 with a Neural Network classifier achieved an optimal balance of high accuracy of 97.10% and F1-score of 95.4%, while the Fine KNN classifier paired with ResNet50 features offered the highest recall of 96.20%, a crucial metric for minimizing missed diagnoses. This exceptional performance suggests a powerful synergy: ResNet50 excels at extracting hierarchical and discriminative feature representations, capturing subtle morphological patterns of impaction, while the Fine KNN classifier effectively leverages these well-separated, high-dimensional features, avoiding potential overfitting and enabling rapid, real-time inference.

In object detection, the modified YOLOv11n surpassed YOLOv10, achieving a superior mAP@0.5:0.95 of 85.7% with greater computational efficiency (19.7 vs 28.4 GFLOPs). Notably, both detection models excelled at identifying anatomically distinct patterns such as inverted and buccolingual impactions. However, mesioangular and horizontal subtypes proved more challenging, a limitation attributed to projective ambiguities and overlapping anatomical structures inherent in 2D OPG, which can lead to moderate precision in these categories. When benchmarked against contemporary studies, our framework addresses significant methodological gaps while maintaining competitive performance, as summarized in Table 5. Previous studies have typically focused on singular tasks. For instance, Chindanuruks et al^21^ and Celik^17^ achieved high performance in detection but lacked multiclass angular classification, which is central to surgical planning. Lei et al^27^ focused on segmentation accuracy for maxillary molars, and Güller et al^18^ combined cone-beam computed tomography and OPG data, facing potential scalability issues. In contrast, our work provides a comprehensive solution through two complementary frameworks trained on a substantial dataset of panoramic radiographs, enabling precise six-class subtype identification.

**Table 5:** Benchmark comparison of AI models for third molar analysis on OPGs.

Study	Dataset size	Models used	Accuracy/mAP	Key limitation
Chindanuruks et al^21^	1730 OPGs	YOLOv5	AUC 72.00%-89.00%	Lacked multiclass angular classification
Celik^17^	440 OPGs	Faster R-CNN, YOLOv3	mAP@0.5 is 96.00%	No subtype detection
Lei et al^27^	708 OPGs	YOLOv5x, distillation CNN	F1 = 95.65%	Focused on maxillary molars, not mandibular
Güller et al^18^	546 CBCT/OPG	SqueezeNet, GoogleNet, Inception-v3	84.00%-93.00%	Scalability of multimodal data
Kumbasar et al^28^	546 PR images from 290 patients	Fusion of AlexNet, VGG16, VGG19	Acc. 79.7%-94.1% (for different MM3-IAN relationship tasks)	Focus on spatial relationship classification (MM3-IAN),
Proposed work (detection)	5796 OPGs	YOLOv10/YOLOv11n	mAP@ (0.5:0.95) 85.70%	Moderate precision for angular subtypes
Proposed work (classification)	5796 OPGs	ResNet50, InceptionNetV3	Acc. 97.56%, 97.33%	Relies on 2D imaging

The clinical implications of this work are substantial. The models’ high performance across diverse impaction types enables reliable presurgical risk stratification, potentially reducing complications like IAN injury. By offering both classification and detection pathways, our approach accommodates varying clinical workflows, from retrospective analysis to real-time diagnostic support. The integration of MHSA and genetic algorithm optimization proved instrumental, enabling the models to focus on clinically relevant features while maintaining the computational efficiency essential for deployment.

## Limitations and future work

Despite its promising results, this study has several limitations. First, the moderate precision in detecting horizontal and mesioangular impactions highlights the inherent challenges of projective ambiguities in 2D panoramic radiographs. Future work should incorporate 3D cone-beam computed tomography data to resolve these spatial uncertainties. Second, while the proposed frameworks are robust, their performance requires further validation across heterogeneous imaging protocols and multicentre datasets to ensure broad generalizability. Finally, exploring more advanced architectures, such as end-to-end transformer-based models, or developing an integrated system that synergistically combines the high accuracy of the classification pipeline with the spatial localization of the detection framework, represents a promising direction for future research.

## Clinical significance

The clinical significance of this work lies in its potential to transform presurgical planning by providing a fully automated, real-time decision-support system. By delivering accurate, subtype-specific classifications from a multinational, expertly annotated dataset, the framework enhances diagnostic consistency across diverse populations and reduces interpretive workload. The improved detection of complex impactions through advanced attention mechanisms and balanced training directly supports safer surgical interventions, allowing surgeons to anticipate complications more effectively. Ultimately, this interpretable and scalable system complements clinical expertise, aiming to reduce the incidence of IAN injury through earlier and more precise risk prediction, thereby improving patient outcomes and standardizing care in oral and maxillofacial surgery.

## Conclusion

This research establishes that both proposed frameworks, deep feature-based classification and optimized YOLO detection architectures, achieve diagnostically superior performance for third molar impaction analysis. The comprehensive evaluation demonstrates that the Fine KNN classifier with ResNet50 features achieved the highest accuracy of 97.56%, while maintaining a precision of 96.07% and a recall of 96.21%. For real-time detection applications, the modified YOLOv11n emerged as the preferred architecture due to its superior precision-recall trade-off, 88.90% mAP@0.5, and computational efficiency (19.7 GFLOPs). The successful integration of MHSA mechanisms and genetic algorithm optimization enabled enhanced feature representation and parameter tuning, respectively. This study provides clinicians with multiple automated options for impaction assessment, potentially improving presurgical planning and reducing complication risks. Future integration with electronic health records and validation through multicentre clinical trials will further enhance the framework’s utility in routine dental practice.

## Author contributions

Zohaib Khurshid: Conceptualization, methodology, validation, formal analysis, investigation, project administration, writing – original draft. M.H.A., F.A.A., A.O.A., A.A.A.: Methodology, formal analysis, investigation, writing – original draft. H.A.A., B.A.A., A.N.A.: Methodology, validation, formal analysis, investigation, writing – original draft.

## Declaration of generative AI and AI-assisted technologies in the writing process

During the revision of this work, the authors used ChatGPT-4 to improve the English language in a few paragraphs, but not the entire manuscript. After using this tool, the authors reviewed and edited the content as needed and took full responsibility for the content of the publication.

## Funding

This work was supported by the Deanship of Scientific Research, Vice Presidency for Graduate Studies and Scientific Research, King Faisal University, Saudi Arabia (Grant No. KFUCreativity-14).

## Data availability

The multinational dental OPG dataset used in this study is available on Mendeley Data.^22^^,^^23^

## Code availability statement

The annotated dataset prepared for this project, corresponding code of the model, and detailed evaluation results are provided in the Mendeley data repository.^26^

## Conflict of interest

The authors declare that they have no known competing financial interests or personal relationships that could have appeared to influence the work reported in this article. The author is an Editorial Board Member/Editor-in-Chief/Associate Editor/Guest Editor for this journal and was not involved in the editorial review or the decision to publish this article.

## Appendix Supplementary materials

Image, application 1

## Footnotes

Supplementary material associated with this article can be found in the online version at doi:10.1016/j.identj.2026.109430.
