# Supplementary material for: Dual Framework for Classification and Detection of Third Molar Impaction in Panoramic Radiographs
Source: Int Dent J. 2026 Feb 9;76(2):109430. doi: 10.1016/j.identj.2026.109430 (PMC12907845; doi:10.1016/j.identj.2026.109430)
Supplement: Supplementary file 1 [file mmc1.docx]

**Supplementary table-1: Checklist for Artificial Intelligence in Medical Imaging (CLAIM) for the third molar Impactions study.**

| **Section / Topic** | **No.** | **Item** | **Page Reference** | **Included?** |
| --- | --- | --- | --- | --- |
| TITLE / ABSTRACT | 1 | Identification as AI study (deep learning) | Page 1 | Yes |
|  | 2 | Summary of design/methods/results/conclusions | Page 1 | Yes |
| INTRODUCTION | 3 | Clinical background & AI's intended role | Pages 2-4 | Yes |
|  | 4 | Study aims/objectives/hypotheses | Page 4 | Yes |
| METHODS: Study Design | 5 | Retrospective study design | Pages 4-5 | Yes |
|  | 6 | Multi-class classification goal | Pages 4-5 | Yes |
|  | 7 | Data sources | Page 10 | Yes |
|  | 8 | Inclusion/exclusion criteria | Pages 6-7 | Yes |
|  | 9 | Preprocessing | Pages 10-11 | Yes |
|  | 10 | Data subset selection | Page 10 (Table 1) | Yes |
|  | 11 | De-identification methods | Page 6 | Yes |
|  | 12 | Missing data handling | Page 6 | Yes |
|  | 13 | Image acquisition protocol | Page 10 | Yes |
| REFERENCE STANDARD | 14 | Clinician annotations (κ = 0.92) | Page 10 | Yes |
|  | 15 | Rationale for reference standard | Pages 6, 10 | Yes |
|  | 16 | Annotation source | Page 10 | Yes |
|  | 17 | Test set annotation | Page 10 | Yes |
|  | 18 | Inter-rater variability | Page 10 | Yes |
| DATA PARTITIONS | 19 | Training/validation/test split | Page 10 (Table 1) | Yes |
|  | 20 | Disjoint patient-level partitions | Page 10 | Yes |
| TESTING DATA | 21 | Intended sample size (n=5,796) | Page 10 | Yes |
| MODEL | 22 | YOLOv11n + ResNet50 + YOLOv10 + InceptionNetV3 | Pages 11-14 | Yes |
|  | 23 | Software: TensorFlow/Albumentations | Page 11 | Yes |
|  | 24 | Parameter initialization | Pages 12-13 | Yes |
| TRAINING | 25 | Loss function/hyperparameters | Pages 12-13 | Yes |
|  | 26 | Final model selection | Pages 15-16 | Yes |
|  | 27 | Feature extraction methods | Pages 13-14 | Yes |
| EVALUATION | 28 | Performance metrics | Pages 14-15, 17-18 | Yes |
|  | 29 | Statistical significance | Pages 18-19 | Yes |
|  | 30 | Robustness analysis | Pages 18-19 | Yes |
|  | 31 | Internal validation | Pages 15-19 | Yes |
|  | 32 | External validation | Not included | No |
| CLINICAL TRIAL | 33 | Clinical trial registration | Not applicable | N/A |
| RESULTS | 34 | Included/excluded cases | Page 10 (Table 1) | Yes |
| MODEL PERFORMANCE | 35 | Metrics with 95% CI | Pages 15-19 | Yes |
|  | 36 | Diagnostic estimates | Pages 15-19 | Yes |
|  | 37 | Confusion matrices | Page 17 | Yes |
| DISCUSSION | 38 | Limitations | Page 22 | Yes |
|  | 39 | Clinical implications | Pages 22-23 | Yes |
|  | 40 | Data/code availability | Page 23 | Yes |
|  | 41 | Funding sources | Page 23 | Yes |
